# Harnessing the protective potential of HIV-1 neutralizing antibodies

**DOI:** 10.12688/f1000research.7254.1

**Published:** 2016-01-05

**Authors:** S Abigail Smith, Cynthia A Derdeyn

**Affiliations:** 1 Department of Pathology and Laboratory Medicine, Emory University, Atlanta, Georgia, 30322, USA; 2 Yerkes National Primate Research Center, Atlanta, Georgia, 30322, USA

**Keywords:** human immunodeficiency virus type 1, HIV-1 neutralizing antibodies, broadly neutralizing antibody, antibody-mediated protection, antibody-mediated therapy

## Abstract

Recent biological, structural, and technical advances are converging within the HIV-1 vaccine field to harness the power of antibodies for prevention and therapy. Numerous monoclonal antibodies with broad neutralizing activity against diverse HIV-1 isolates have now been identified, revealing at least five sites of vulnerability on the envelope (Env) glycoproteins. While there are practical and technological barriers blocking a clear path from broadly neutralizing antibodies (bNAb) to a protective vaccine, this is not a dead end. Scientists are revisiting old approaches with new technology, cutting new trails through unexplored territory, and paving new roads in the hopes of preventing HIV-1 infection. Other promising avenues to capitalize on the power of bNAbs are also being pursued, such as passive antibody immunotherapy and gene therapy approaches. Moreover, non-neutralizing antibodies have inhibitory activities that could have protective potential, alone or in combination with bNAbs. With a new generation of bNAbs, and a clinical trial that associated antibodies with reduced acquisition, the field is closer than ever to developing strategies to use antibodies against HIV-1.

## Introduction

Neutralizing antibodies against HIV-1 were first described early in the HIV-1 epidemic ^1^, raising hopes that a protective vaccine was in the immediate future. Yet 30 years later, only one vaccination regimen has shown any efficacy in humans to date ^2^, essentially in the absence of functional neutralizing antibody activity ^3, 4^. Indeed, most vaccine approaches tested so far mainly elicit antibodies that can only neutralize T-cell line-adapted strains and the minor fraction of naturally occurring strains that exhibit a highly sensitive tier 1 neutralization phenotype ^4– 8^. This is because most naturally occurring HIV-1 isolates tend to mask vulnerable surfaces, making them much more difficult to neutralize, classified as possessing a tier 2 or 3 phenotype ^9– 11^. Stopping these neutralization-resistant, genetically diverse HIV-1 strains, which constitute the majority of the current global pandemic, will require an approach that successfully targets one or more regions of the trimeric envelope glycoprotein (Env) spike that are vulnerable to neutralization on most tier 2 viruses. However, all broadly neutralizing antibodies (bNAbs) identified thus far possess one or more unusual features ^12^, suggesting that they will be difficult to generate via vaccination. To overcome this and other potential obstacles, the HIV-1 research community is now moving forward along two major paths: (1) developing novel strategies to elicit antibodies capable of broadly and potently neutralizing clinically relevant, genetically diverse HIV-1 strains and (2) combining knowledge of bNAbs with modern technologies to create protective alternatives and therapeutic options ( Figure 1).

**Figure 1.**
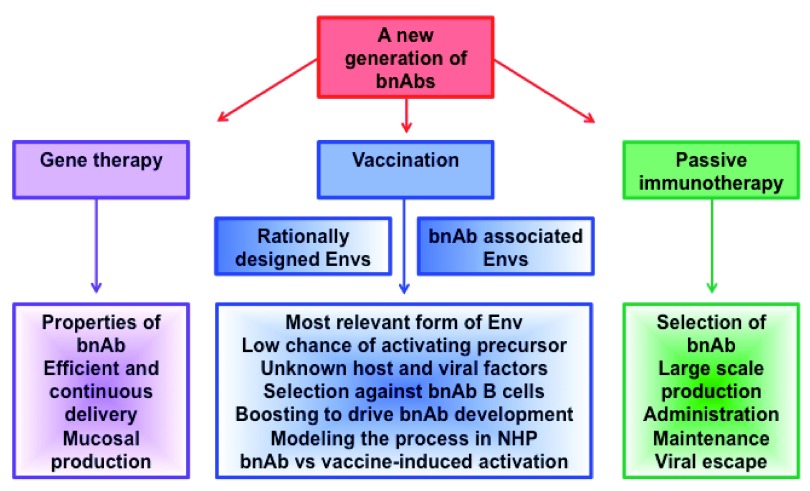
Three promising avenues for using HIV-1 broadly neutralizing antibodies (bNAbs) for protection and therapy. Each area of pursuit is shown, and potential obstacles are listed below. For vaccination, there are two approaches for generating immunogens that will face common barriers. Env, envelope glycoprotein; NHP, non-human primate.

## Innovative application of bNAbs for HIV prevention and therapy

During natural HIV-1 infection, most individuals develop antibodies with some level of heterologous neutralization breadth ^13, 14^, although only a small fraction of these individuals go on to develop high titers of the most extraordinary bNAbs ^15^. A focused effort to recover monoclonal antibodies from these rare individuals with very potent and broad plasma-neutralizing activity has led to a collection of a few dozen bNAbs, defined as having the ability to neutralize more than 60% of the variants in multi-clade HIV-1 Env panels at a concentration of less than 50 μg/ml ^12, 16^. In turn, these discoveries have led to the identification of five regions on the viral Env trimer that are accessible to bNAbs: the CD4-binding site (CD4bs), the gp120 V1V2 hyper-variable domain, a glycan patch centered on the base of the gp120 V3 domain, the gp120 and gp41 interface, and the membrane proximal external region (MPER) of gp41. These discoveries have collectively bolstered optimism that bNAbs could also be elicited by vaccination.

However, genetic and structural characterizations of the most promising bNAbs have revealed substantial obstacles to eliciting them through vaccination. Although HIV-1 bNAbs can arise from several immunoglobulin germline precursors, they have unusual features such as high levels of somatic hypermutation, uncharacteristically long CDR H3 (complementarity determining region H3) domains, and in some cases polyreactivity ^12^. This may be because bNAbs must recognize complex epitopes that exhibit variation across HIV-1 strains and are often composed of both glycan and peptide ^17– 21^. Moreover, HIV-1 Env protein immunogens often do not readily bind and activate the germline precursors that have been associated with bNAb development, complicating immunogen design ^22– 26^. To gain more insight into the ontogeny of bNAbs, recent studies identified individuals who developed neutralizing antibody breadth early in infection and tracked co-evolution of the autologous virus and antibody lineages ^27– 30^. Key features that drive bNAb development appear to be the presence of a specific antibody germline precursor, a viral Env that can engage it, and temporal exposure of the antibody lineage to an array of highly related Env variants presenting different versions of the same epitope. The proficiency with which an antibody can neutralize autologous escape variants seems to be directly correlated with its ability to neutralize genetically diverse isolates ^29^. Yet even within a successful bNAb lineage, there are antibodies that share the features associated with bNAb activity but do not acquire breadth ^29, 31, 32^. The observation that some antibodies follow an evolutionary path toward acquiring neutralization breadth, while others do not, has yet to be explained. Incorporating information from these longitudinal studies of bNAb development into the design of Env immunogens that mimic this naturally occurring, but rare, process will be challenging, since it is likely that other host and viral factors also influence bNAb development ^12, 33^.

Now that the development of two bNAb lineages has been traced in natural infection and the initiating and mutated Env variants determined, panels of Env immunogens based on these pathways can be developed and tested. Then again, the most important considerations for evaluating these novel Env immunogen panels are ambiguous. Should the indication to move forward with a series of immunogens be marked by successful stimulation of a particular bNAb germline, or a bNAb-related specificity with any germline? What level of heterologous tier 2 neutralization should be considered a success? Should protection from viral challenge also be demonstrated? Although transgenic mice have been used to model bNAb induction ^34, 35^, the rhesus macaque model remains the most relevant for HIV vaccine studies. But this model is not ideal. There is limited information about the development of neutralization breadth in HIV-1 Env-immunized or chimeric simian-human immunodeficiency virus (SHIV)-infected rhesus macaques ^36– 40^, and the antibody germline repertoire of this species is similar but not identical to that of humans ^39, 41^. Interestingly, Env-specific antibodies from immunized rhesus macaques that exhibited the highest somatic hypermutation levels arose from a single VH (immunoglobulin variable domain heavy-chain segment) gene ^37^. Furthermore, a high level of somatic hypermutation in VH was not associated with serum neutralization breadth. Studies of immunized rhesus macaques have also demonstrated that CD4bs antibodies can be elicited, but these antibodies do not exhibit breadth, possibly because their angle of approach differs from that of the VRC01-like CD4bs bNAbs ^42, 43^. Thus, it will be important to carefully consider similarities and differences among non-human primates and humans when evaluating strategies to elicit bNAbs.

Another uncertainty regarding testing naturally occurring bNAb-associated Env immunogens is what form of Env should be administered. Do immunogens that retain the native trimeric Env structure present more relevant epitopes than monomeric gp120 or gp140 proteins for eliciting antibodies with breadth? The best-characterized Env trimer to date, BG505 SOSIP.664, is capable of activating inferred germlines from bNAbs *in vitro* ^26^. However, thus far, immunization of rhesus macaques with the BG505 SOSIP.664 trimer has not induced cross-reactive tier 2 neutralizing antibody responses ^8^. The BG505 trimer immunogen did elicit low titers of antibodies that neutralized the tier 2 autologous pseudovirus and higher titers of antibodies that neutralized tier 1 Env variants. In addition, rhesus macaques immunized with a different trimer produced an antibody response that was polyclonal even within those directed against a single target ^42^. Additional Env trimers from different HIV-1 genetic subtypes (clades) of the virus are being generated and characterized ^44^ and this will lead to a greater selection of immunogens to build upon.

Another matter to consider is that a precursor B cell that is capable of initiating a bNAb lineage may be relatively rare within the immune repertoire. To highlight the low chance of activating a particular heavy chain (VH) germline, there are approximately 76 to 84 functional VH possibilities in the human genome. The VH3-30 family, which is found in the V1V2-targeted bNAb lineage CAP256-VRC26 described by Doria-Rose *et al.* ^27^ and Bhiman *et al.* ^29^, contains 19 different alleles ^45^. Furthermore, there are inherent biases for and against heavy chain families and even individual alleles within families. Stimulating a specific B cell out of so many possibilities, in a genetically diverse population, is a daunting task. Compounding the rarity of the initial B cell, even the unmutated common ancestor, may need to have acquired unique features, such as a long CDR H3, or unusual insertions/deletions. The CAP256-VRC26 bNAb lineage was initiated by an unmutated precursor that had an extraordinarily long CDR H3 region ^27, 29^. At 35 to 37 amino acids, the CAP256-VRC26 CDR H3 regions are more than twice the average length for human antibodies, approximately 15 amino acids ^46^. These inherent obstacles will need to be taken into consideration when designing a vaccine strategy. Finally, a specific Env variant that is capable of activating this unique germline must be present. It seems unlikely that a randomly selected Env immunogen will elicit a bNAb lineage. In the case of CAP256-VRC26, a variant that evolved from a superinfecting virus, but not the primary infecting virus quasispecies, initiated the bNAb lineage ^29^. The site of recognition on the antigen(s) by that initial antibody precursor also seems to be important, as different pathways to neutralization breadth were described for V1V2- and CD4bs-targeted bNAbs, the latter of which required a second helped antibody lineage ^27, 28^.

Even with more knowledge about bNAbs and how they develop, the barriers standing in the way of an HIV vaccine are formidable. Avenues that are being explored independently of a vaccine are passive administration of bNAbs for immunotherapy and prevention. With a newer generation of bNAbs, an older idea such as passive immunotherapy might be more effective. The process of selecting bNAbs to advance into the clinical pipeline will require a careful assessment of multiple antibody properties, including their stabilities, pharmacokinetic profiles, and safety. One phase I trial of bNAb VRC01, which targets the CD4bs, has been conducted evaluating both intravenous and subcutaneous administration. This study indicated that both delivery routes were safe; however, subcutaneous delivery may be limited by the dosing required for adults and may be more suitable for infants ^47^. Caskey *et al.* infused variable doses of 3BNC117, which also targets the CD4bs, into healthy controls and HIV ^+^ patients ^48^. This monoclonal antibody therapy was well tolerated, and all of the HIV ^+^ patients who received the highest dose experienced significant decreases in viral load. Predictably, in some patients, a familiar specter emerged: mutations present within the patients’ viral quasispecies facilitated escape from 3BNC117 neutralization. Thus, like the scenario that successful antiretroviral drug therapy requires the use of several different inhibitors, passive immunotherapy will likely require the use of a cocktail of bNAbs that target distinct regions of Env. An additional consideration is that bNAbs do not always exhibit complete neutralization *in vitro*. In some cases, a subfraction of virions exists that is refractory to inhibition. This “incomplete neutralization” phenomenon has been attributed to heterogeneity in virion-associated Env glycosylation, or conformation within the genetically clonal virus population, or both ^49, 50^. bNAbs that are more tolerant of this type of variation are more likely to achieve complete neutralization, but this is also dependent upon the properties of the Env variant itself ^49^. Recently, for bNAbs that are capable of complete viral neutralization, it was shown that the slope of the neutralization curve could be an important determinant of the therapeutic potential of a particular bNAb and should be considered along with breadth and potency ^50^. This study also revealed that CD4bs and V3 base/glycan-targeted bNAbs, because of their high inhibitory potential, could be especially beneficial for immunotherapy.

Besides viral escape, other roadblocks to the successful and widespread implementation of passive immunotherapy are the cost and logistics associated with the mass production of a sufficient amount of bNAbs. Researchers are now using tobacco plants to produce an arsenal of HIV-1 bNAbs ^51, 52^. The tobacco plants have been genetically modified to modulate antibody glycosylation and sulfation and allow antibody production to be ramped up to an industrial scale. A cocktail of monoclonal antibodies produced in this manner was used as an emergency treatment for Ebola during the 2014 epidemic ^53, 54^. With the recovery of so many different HIV-1 bNAbs, some with extraordinary potency and breadth, immunotherapy could also have an important role to play in treating HIV-1 infection.

Given the uncertainty of whether bNAbs can be induced via a vaccine, researchers are also venturing into the uncharted territories of gene therapy, which combined with the new generation of bNAbs has opened up new options. Gene therapy protocols are being developed to potentially deliver bNAb over long periods of time; the idea is that a sufficient amount of bNAbs present at the time of exposure might be able to prevent HIV-1 infection altogether. This concept is supported by numerous observations that passive administration of bNAbs to rhesus macaques at high concentrations can prevent mucosal SHIV infection ^55^. One question that remains is whether bNAbs generated by passive administration, gene therapy, and immunization will all have the same protective potential ^56– 59^. As proof of concept, adeno-associated virus (AAV) has recently been used as a vector to deliver the genetic material that encodes VRC07, a CD4bs bNAb, into humanized mice and rhesus macaques. Both animal models showed that this mode of antibody delivery could protect from a mucosal challenge ^60, 61^. Gardner *et al.* took this concept a step further, creating a novel chimeric antibody that is more potent and broad than even the best bNAbs ^62^. This construct, eCD4-Ig, combines the broad recognition ability of CD4 (a receptor essential for all HIV-1, HIV-2, and simian immunodeficiency virus [SIV] variants) with a sulfated peptide that mimics the co-receptor CCR5. When AAV was used to deliver eCD4-Ig to rhesus macaques, the animals showed no sign of infection for the duration of the study (40 weeks post-therapy), despite escalating intravenous challenges. This represents a stellar example of how a strong basic research program can lead to innovative and promising approaches ^63– 67^.

Given the difficulties in delivering bNAbs discussed above or in eliciting them by vaccination, some researchers are staying the course to a vaccine by developing approaches to protect against HIV-1 without bNAb. One alternative is to generate antibodies capable of mediating antibody-dependent cellular cytotoxicity (ADCC). Although the RV144 vaccine trial generated antibody responses with minimal neutralization capabilities, these same antibodies were capable of mediating ADCC ^68^. Thus, it is worth revisiting the protective potential of non-neutralizing antibodies, which could be more amenable to elicitation by a vaccine ^57, 69– 71^. Another option is to delve into the antibody ontogeny of more HIV-1-infected patients, looking for more attainable antibody goals as well as identifying viral and immunological roadblocks that prevent the development of neutralization breadth. Rather than trying to coax the immune system into making rare antibodies, one could focus on strategies to elicit antibodies with moderate breadth, limited somatic hypermutation, typical CDR H3 lengths, and commonly used VH germline precursors ^72^. Perhaps eliciting combinations of these more common types of antibodies would provide some level of protection in the end.

## Summary

The past several years have seen major advances in terms of antibody-mediated protection and therapy against HIV-1. bNAbs, though rare, are generated in multiple HIV-1-infected individuals. Insight into bNAb ontogeny during natural infection has revealed novel strategies for vaccination, but at the same time these studies have highlighted the difficulties that will need to be overcome. Other avenues for passive therapy and protection have become possible because of the new generation of bNAbs, some of which can neutralize up to 90% of genetically diverse isolates tested and could be even more effective when combined optimally ^73^. Innovative design of Env immunogens, chimeric antibodies, and enhanced bNAbs may also yield protective or therapeutic benefits. Finally, bNAb-independent approaches that involve more moderate neutralization breadth or non-neutralizing effector functions may also hold promise.

## Abbreviations

AAV, adeno-associated virus; ADCC, antibody-dependent cellular cytotoxicity; bNAb, broadly neutralizing antibody; CD4bs, CD4-binding site; CDR H3, complementarity determining region H3; Env, envelope glycoprotein; SHIV, chimeric simian-human immunodeficiency virus; V1V2, gp120 hyper-variable domains 1 and 2; V3, gp120 variable domain 3; VH, immunoglobulin variable domain heavy-chain segment.
